# Characterization of Neutral Lipase BT-1 Isolated from the Labial Gland of *Bombus terrestris* Males

**DOI:** 10.1371/journal.pone.0080066

**Published:** 2013-11-08

**Authors:** Jana Brabcová, Darina Prchalová, Zuzana Demianová, Alena Bučánková, Heiko Vogel, Irena Valterová, Iva Pichová, Marie Zarevúcka

**Affiliations:** 1 Institute of Organic Chemistry and Biochemistry AS CR, Prague, Czech Republic; 2 Agricultural Research, Ltd., Troubsko, Czech Republic; 3 Max Planck Institute for Chemical Ecology, Jena, Germany; Ghent University, Belgium

## Abstract

**Background:**

In addition to their general role in the hydrolysis of storage lipids, bumblebee lipases can participate in the biosynthesis of fatty acids that serve as precursors of pheromones used for sexual communication.

**Results:**

We studied the temporal dynamics of lipolytic activity in crude extracts from the cephalic part of *Bombus terrestris* labial glands. Extracts from 3-day-old males displayed the highest lipolytic activity. The highest lipase gene expression level was observed in freshly emerged bumblebees, and both gene expression and lipase activity were lower in bumblebees older than 3 days. Lipase was purified from labial glands, further characterized and named as BT-1. The *B. terrestris* orthologue shares 88% sequence identity with *B. impatiens* lipase HA. The molecular weight of *B. terrestris* lipase BT-1 was approximately 30 kDa, the pH optimum was 8.3, and the temperature optimum was 50°C. Lipase BT-1 showed a notable preference for C8-C10 *p*-nitrophenyl esters, with the highest activity toward *p*-nitrophenyl caprylate (C8). The Michaelis constant (K_m_) and maximum reaction rate (V_max_) for *p*-nitrophenyl laurate hydrolysis were K_m_ = 0.0011 mM and V_max_ = 0.15 U/mg.

**Conclusion:**

This is the first report describing neutral lipase from the labial gland of *B. terrestris*. Our findings help increase understanding of its possible function in the labial gland.

## Introduction

Lipases control the key steps in lipid uptake, transport, and utilization in many animal species [1]. Lipase activity has been reported in the insect reproductive system [2] and in other insect tissues [3,4]. The first lipase purified from an insect was a triacylglycerol-lipase from *Manduca sexta* fat body [5]. Recent studies have focused on identification, characterization, and endocrine control of lipases in insect fat bodies [6-8] and identification of phospholipase from venom glands of *Apis mellifera* and *Nasonia vitripennis* [9-11].

In bumblebees, lipases from the cephalic part of the labial pheromone glands (LGs) might be involved in the biosynthesis of male marking pheromones. Male marking pheromones are species-specific blends of numerous compounds (most often esters, alcohols, terpenes, and hydrocarbons) [12,13]. In *B. terrestris*, the marking pheromone consists of isoprenoids (2,3-dihydrofarnesal, 2,3-dihydrofarnesol, geranylcitronellyl butyrate, geranylcitronellol, 2,3-dihydrofarnesyl tetradecenoate), fatty alcohols (hexadecanol, octadecatrienol, octadecenol, icosenol, docosenol), higher alkanes (henicosane, tricosene, tricosane, pentacosane, heptacosene, nonacosene), and esters (ethyl dodecanoate, hexadecyl dodecanoate) [13,14]. There are two hypothetical biosynthetic pathways leading to fatty acid pheromone precursors in the cephalic part of LGs: 1) release of fatty acid precursors from diacylglycerols by a specific LG lipase, and 2) *de novo* synthesis of fatty acids from acetate units in the LGs themselves. 

We hypothesized that a specific lipase associated with LGs in bumblebee species might modify fatty acids released from storage triacylglycerols (TAG), converting them into pheromone components in a manner similar to that reported in some moths [15]. By incubating LGs from *B. terrestris* with labeled acetate *in vitro*, we showed that pheromone precursors can be synthesized *de novo* [16]; however, our results did not exclude the alternative formation of pheromone precursors from pooled lipids [17]. In this alternative pathway, the fatty acid pheromone precursors are likely released from their transported form (diacylglycerols) by selective lipases. In the present study, we identified lipase BT-1 from the labial gland of *B. terrestris* males and monitored the temporal dynamics of its enzymatic activity. The enzyme’s substrate specificities towards *p*-nitrophenyl esters with varying alkyl chain lengths and triolein were investigated. The enzyme’s pH optimum, thermal stability, kinetic parameters, and molecular weight also were determined. This information helps increase understanding of the function of this lipase in the LG of *B. terrestris* males.

## Materials and Methods

### Insects

*B. terrestris* males of various ages were obtained from laboratory colonies during the 2010–2011 winter season, as previously described by Ptáček [18]. 

### Preparation of crude enzyme mixture

LG tissues from 60 decapitated bumblebees were collected in ice-cold homogenization buffer (25 µl per organ): 20 mM Tris-HCl, pH 7.4, containing 0.25 M sucrose, 1 mM NaEDTA, 0.1 mM benzamidine, 0.1% (v/v) 2-mercaptoethanol, 10 mg/l leupeptin, and 1 mg/l aprotinin [5]. The tissues were homogenized using a Potter-Elvehjem homogenizer with a Teflon pestle. The homogenate was centrifuged at 20,000 x g for 20 min. The floating fat cake was removed, and the supernatant was transferred into Eppendorf tubes. The pellet was resuspended in homogenization buffer (25 μl per LG) and centrifuged at 20,000 x g for 20 min. The supernatants were collected and centrifuged at 20,000 x g for 30 min. All steps were carried out at 4°C. Glycerol was added to a final concentration of 50% (w/v), and the suspension was stored at -20°C until use.

### Purification of lipases

Octyl-agarose (1 g) was added to 20 ml crude extract prepared from 60 bumblebee LGs. The mixture was dialyzed against 10 mM phosphate buffer (pH 7.8) and then continuously shaken overnight at 25°C. Octyl-agarose with adsorbed protein was washed with distilled water, and proteins were desorbed by step-wise treatment with various concentrations of Triton X-100 (ranging from 0.1–1%) in 10 mM sodium phosphate buffer, pH 7.8. During each step, the mixture was shaken for 30 min at 25°C, and desorbed proteins were collected. Between each step, the support was washed with distilled water. The major active protein, lipase RT-1, was desorbed from octyl-agarose with 0.2 % Triton X-100. Detergent was removed by ultrafiltration using Amicon filters.

### Lipase activity assay and determination of substrate specificity

Enzyme activity was evaluated as described by Vorderwülbecke [19], with a slight modification. The reaction mixture contained 1 ml homogenization buffer, 100 μl substrate (3 mM *p*-nitrophenyl ester in 2-propanol), and 40 μl purified enzyme To evaluate the enzyme’s hydrolytic specificity for various substrates, its activity was studied with chromogenic *p*-nitrophenyl ester substrates with varying alkyl chain lengths: *p*-nitrophenyl caprylate (C8, *p*-NPC), *p*-nitrophenyl decanoate (C10, *p*-NPD), *p*-nitrophenyl laurate (C12, *p*-NPL), *p*-nitrophenyl palmitate (C16, *p*-NPP), and *p*-nitrophenyl stearate (C18, *p*-NPS). The reaction was started by addition of the substrate. Release of *p*-nitrophenol was monitored spectrophotometrically at 410 nm. One unit of enzyme activity (U) was defined as the amount of enzyme releasing 1 µmol of *p*-nitrophenol per min under the given experimental conditions. Specific activity was expressed as U/mg protein. The concentration of proteins in the crude extract was determined by Bradford assay using bovine serum albumin as a standard [20].

### Determination of lipase kinetics

The effect of *p*-NPL concentration (ranging from 0.05 to 0.4 mM in 2-propanol) on the reaction rate was determined at 25°C in 50 mM Tris/HCl buffer, pH 8.2. The Michaelis constant (K_m_) and the maximum activity rate (V_max_) were determined from a Lineweaver-Burk plot.

### Activity assay with ^14^C-labeled triolein

We also used a previously described radiometric assay [3,21] to measure lipase activity. This assay employed ^14^C-labeled triolein (the isotope was present at the carboxyl carbon of the oleic acid moiety esterified to each of the three glycerol carbons) as a substrate, and activity was measured as radioactivity recovered in free fatty acid after exposure to lipase. A stock solution of radiolabeled triolein was obtained from New England Nuclear (3.7 MBq/ml). Stock solution (50 µl) was added to buffer so that the final reaction mixture contained: 150 mM [^14^C] triolein, 1% (w/v) fatty-acid-free bovine serum albumin, 50 mM Tris-HCl, pH 8. Reactions were initiated by the addition of 50 µl of purified enzyme to 175 µl of the final reaction mixture buffer. Immediately following addition of the enzyme, a small aliquot (50 µl) of the reaction mixture was sampled for the determination of specific radioactivity. The reaction was held at 25°C with continuous shaking. Reactions were stopped by the addition of extraction mixture [chloroform:methanol:benzene (1:2.4:2 by volume), 1 ml], and free fatty acids were extracted. The pH of the mixture was then adjusted to 11.5 by addition of 0.1 M NaOH (40 µl), and the aqueous and organic solvent phases were separated by centrifugation at 1,000 x g for 10 min. An aliquot (60 µl) of the upper aqueous phase containing de-esterified oleic acid was removed for determination of radioactivity. Radioactivity was determined with a Tri-Carb 2900TR liquid scintillation spectrometer. The concentration- and time-dependence of the reaction were assessed. Lipase activity was expressed as pmoles of [^14^C] oleic acid released per min per mg protein.

In order to study the effect of triacylglycerols and diacylglycerols on hydrolysis of ^14^C-labeled triolein, the reaction mixture was prepared by diluting the triolein stock solution described above in buffer so that the final reaction mixture contained: 125 mM [^14^C] trioleate, 1% (w/v) fatty-acid-free bovine serum albumin, 25 mM Tris-HCl, pH 7.5, and 25 mM non-radiolabeled competing substrate. The total triacylglycerol concentration in the assay was 150 mM, comprised of 83.3% radiolabeled trioleate and 16.6% nonradioactive TAG [trioleate in the case of control experiments, or other species of TAG (trimyristate, tripalmitate, tristearate) in the case of competition experiments] or nonradioactive diacylglycerols (dipalmitate, dioleate, dilaurate). Reactions were carried out at 25°C.

### Effects of pH and temperature on lipase activity

The effect of pH on enzyme activity was determined by performing activity assays in various buffers with different pH ranges. The following 50 mM buffers were used: Gly-HCl buffer (pH 2.2 and 2.8), sodium citrate buffer (pH 3.2 - 6.2), sodium phosphate buffer (pH 6.5 and 7.0), Tris-HCl buffer (pH 7.6 - 8.8) and Gly-NaOH buffer (pH 9.0 -11.0). To determine the optimum pH of lipase activity, 40 µl purified enzyme was pre-incubated at different pHs (pH 2.2 - 11.0) for 10 min at 25°C. The reaction was started by addition of the substrate (100 µl 3 mM *p*-NPL in 2-propanol), and the enzymatic activity was measured spectrophotometrically at 410 nm. 

To determine the effect of temperature on lipase activity, 20 µl of enzyme was pre-incubated in 50 mM Tris-HCl buffer (1 ml, pH 8) for 5 min at different temperatures (4°C - 70°C). The reaction was started by addition of the substrate (100 µl 3 mM *p*-NPL in 2-propanol), and the enzyme activity was measured spectrophotometrically at 410 nm. 

### Effects of detergents on lipase activity

Lipase (30 µl) or homogenization buffer was added to 1 ml of homogenization buffer containing various concentrations of detergents [0.01 - 0.25% (w/v) Triton X-100, Tween 20, or Tween 80]. After 10 min incubation at 25°C, the reaction was initiated by quickly shaking the reaction mixture with 100 µl *p*-NPL (3 mM in 2-propanol). Absorbance was recorded at 410 nm.

### Sensitivity to metal ions

Lipase (40 µl) was added to stock solutions containing compounds with potential inhibitory activity (1 ml, final concentrations: 0.1, 1.0, or 10.0 mM) and incubated at 25°C for 1 h. At the end of the incubation period, *p*-NPL was added to the mixture, and residual lipase activity was spectrophotometrically recorded.

### Electrophoresis and preparation of zymograms

Sodium dodecyl sulfate polyacrylamide gel electrophoresis (SDS-PAGE) was performed on vertical slab gels according to Laemmli’s procedure [22], using 5% and 12% polyacrylamide for the stacking and resolving gels, respectively. Broad range molecular weight standards (Protein Test Mixture 6) were used for mass determinations. Proteins were stained with Coomassie Brilliant Blue R250. 

Non-denaturing SDS-PAGE was performed using a discontinuous SDS-PAGE gel under non-reducing conditions (protein samples were not treated with 2-mercaptoethanol or high heat before electrophoresis). Electrophoresis was carried out at 180 mV at 4°C [23]. For lipase activity detection, SDS was removed from the gel by washing once for 20 min with 20% 2-propanol and twice for 10 min with distilled water at 25°C [24]. The gel was briefly washed in 50 mM Tris-HCl, pH 8.0, and covered with a solution of 100 µM methylumbelliferone butyrate (MUF-butyrate) in the same buffer [25]. Activity bands became visible shortly after UV illumination. Following the gel (zymogram) analysis, the separated protein bands were visualized using Coomassie Brilliant Blue R250.

### Mass spectrometry

Identification of lipases was performed using an LC Ultimate 3000 RSLC nano system coupled to a TripleTOF™ 5600 mass spectrometer system. The protein bands were excised from the preparative gels and digested in-gel with trypsin (0.4 µg). Extracted tryptic peptides were concentrated by vacuum centrifugation and acidified with 0.1% formic acid to a final volume of 30 µl. The sample injection volume was 10 µl with a flow rate of 5 µl/min on the trap column (Acclaim PepMap100 C18, 3 µm particles, 75 µm x 2 cm), and the separation was carried out on a PepMap RSCL C18 column (75 µm x 150 mm x 3 µm) using a flow rate of 300 nl/min. The mobile phases consisted of 0.1% formic acid (A) and 0.1% formic acid in 100% acetonitrile (B). A four-step linear gradient of 5–50% B for 55 min, 50-99% B for 5 min, and 99% B for 10 min was used. An ion spray voltage floating of 2400 V was applied with ion gas of 8 abs and curtain gas of 25 abs. The MS scan range was m/z 350 -1,200 in high resolution mode (˃30,000), and the top 25 precursor ions were selected for subsequent MS/MS scans in high sensitivity mode (at least ˃15,000) with rolling collision energy.

All MS/MS spectra were processed using ProteinPilot™ Software 4.0 with integrated false discovery rate (FDR) analysis. The software used only unique peptide sequences as evidence for  protein identification. The collected MS spectra were searched against the NCBI- protein database containing canonical and isoform sequence data from *B. terrestris* (version released in August 2012) or our contig databases, obtained from RNA sequencing, with the following parameters: Sample Type- gel ID; Cys Alkylation- iodoacetamide; Digestion- trypsin; Search Effort- Thorough; Detected Protein Threshold- 0.05 (10.0%). Only peptides identified with ≥ 95% confidence were taken into account for identification. 

### Isolation of the lipase gene

The central fragment of the lipase gene was amplified by PCR using hybrid primers designed with CODEHOP [26] and a bumblebee cDNA library as a template. The primers (lipCOD forward: 5’-CGGAGACTGCAACGTGATCryngtngaytgg-3’, lipCOD reverse: 5’- GCCCAGTAATCCACCGtyngtntgdat-3’) were designed based on the sequence comparison of the lipase genes from several insect species obtained from GenBank (NCBI). They consist of a short 3’ degenerate core region and a longer 5’ consensus clamp region (lower and upper case letters, respectively). The starting material for the cDNA library, which was constructed in a λTriplEx2 vector, was RNA isolated from the labial gland and fat body of a 3-day-old *B. terrestris* male. The purified PCR product was directly cloned into a pCR^®^II-TOPO^®^ vector (Invitrogen) and sequenced. The identity of the sequence obtained was confirmed using BLAST (NCBI). 

To isolate the rest of the lipase gene, Rapid Amplification of cDNA Ends (RACE) PCR reactions with the cDNA library, gene-specific primers (BTlip forward: 5’-CGTTTACGTGTCCAGCCAAG-3’, BTlip reverse: 5’-GATAACAACGGCTTCGGCGT-3’), and primers complementary to the λTriplEx2 vector was performed. The complete sequence of the lipase gene was deposited in the GenBank database under accession number KF006994 and was used to design specific primers for qPCR. 

### Sequence analysis

Protein sequence alignment was performed using Multalin version 5.4.1 (Multiple sequence alignment with hierarchical clustering ([27], http://multalin.toulouse.inra.fr/). Phylogenetic analysis was performed with http://www.phylogeny.fr/version2_cgi/index.cgi [27-29].

### Quantitative real-time PCR

Total RNA (0.25 µg) was isolated from LGs using Trizol (Invitrogen) according to the manufacturer’s protocol. RNA served as a template for cDNA synthesis using SuperScript III Reverse Transcriptase (Invitrogen) and random hexamer primers according to the manufacturer’s procedure. qPCR primers (realBTlip forward: 5’-CGTTTACGTGTCCAGCCAAGTT-3’, real BTlip reverse: 5’-GCCCAAAGAATGCCCAATCA-3’) were designed using Primer 3 [30]. Serial 10-fold dilutions of the pooled cDNA from all samples were analyzed to calculate the amplification efficiency for the primer pair. 

qPCR experiments were performed using a LightCycler^®^ 480 Real-Time PCR System (Roche) with SYBR^®^ Green fluorescent labeling. The reaction mixture consisted of Dynamo™ HS SYBR^®^ Green qPCR Master Mix (Finnzymes), 0.625 µM of each primer, and 1 µl of the appropriate cDNA in a 20 µl reaction volume. The cycling conditions were: 95 °C for 15 min and 45 cycles of 95°C for 30 s, 50°C for 30 s, and 72°C for 30 s. Thermal cycling was followed by a melting-curve analysis of the final PCR products to verify the presence of a single amplicon. All samples were examined in two technical replicates. Data were exported from the LightCycler^®^ 480 SW 1.5 into Microsoft Excel and analyzed using GenEx software (www.multid.se). Relative gene expression was normalized to phospholipase A2 (PLA2) and elongation factor 1α (EEF1A). Primers for PLA2 were PLA2 forward (5’-GGTCACACCGAAACCAGATT-3’) and PLA2 reverse (5’-TCGCAACACTTCGTCATTTC-3’), and primers for EEF1A were EEF1A forward (5’-AGAATGGACAAACCCGTGAG-3’) and EEF1A reverse (5’-CACAAATGCTACCGCAACAG-3’) [31]. 

## Results

### Screening lipase activity in the labial glands of *B. terrestris* males of different ages

In *B. terrestris* males, the cephalic part of the LG produces a large amount of secretion (up to 1 mg per single male), the composition of which changes with age. The secretion begins immediately after eclosion, and production reaches a maximum about 5 days post-eclosion and stops around the 10^th^ day of adult life. 

**Figure 1:**
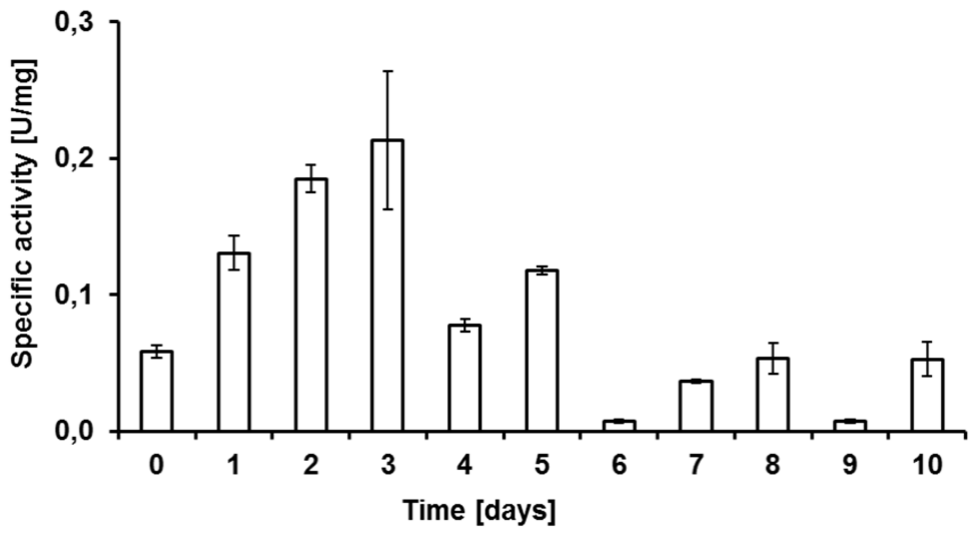
Temporal dynamics of lipolytic activity of enzymes in the crude labial gland extract. The lipase activity was determined using *p*-nitrophenyl laurate as substrate. Release of *p*-nitrophenol was monitored spectrophotometrically at 410 nm. One unit of enzyme activity (U) was defined as the amount of enzyme releasing 1 μmol of *p*-nitrophenol per min under the given experimental conditions.

The temporal dynamics of lipase hydrolytic activity in *B. terrestris* LG tissue (Figure 1) were detected in all stages tested (freshly emerged males to 10-day-old males); activity was normalized according to protein content [*p*-nitrophenol (µmol) per min divided by protein amount (mg)]. Maximal lipase activity was observed in LGs from 3-day-old individuals, which corresponds with the timing of pheromone formation in the LG. 

### Isolation of lipase from the labial glands of *B. terrestris* males

**Figure 2:**
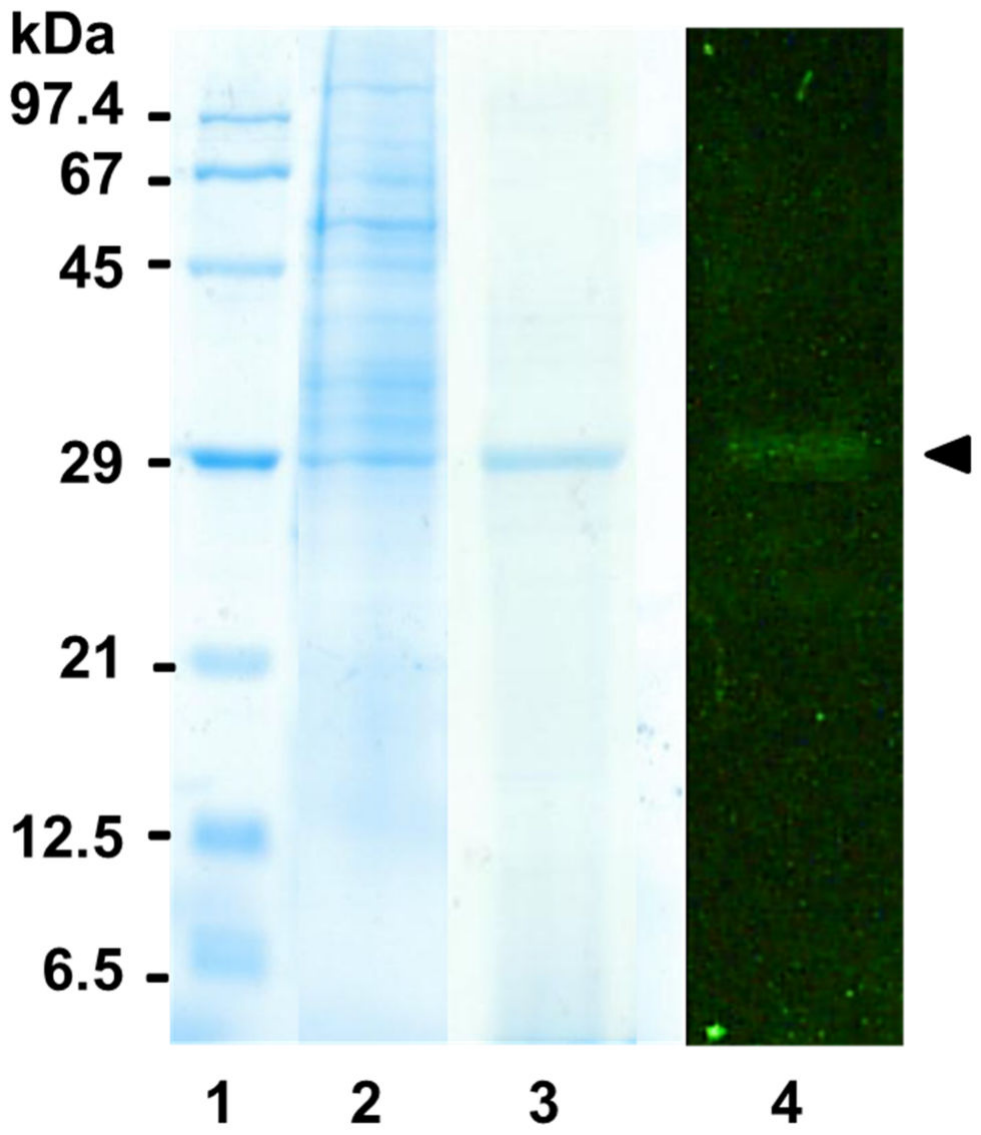
Zymogram analysis from the SDS-PAGE of purified lipase. Samples stained with Coomassie Brilliant Blue R-250 (Lanes 1-3) and analyzed for lipolytic activity using MUF-butyrate (Lane 4). Lane 1: molecular weight standards, Lane 2: crude extract, Lane 3: lipase bound on octyl-sepharose, Lane 4: lipase BT-1 released from the support with 0.2% Triton X-100.

Based on these results, we purified lipase from the LGs of 2- to 3-day-old bumblebees. We used an efficient method involving the interfacial activation of lipases on a hydrophobic support to separate and purify lipases from crude LG extracts [32,33]. SDS-PAGE analysis of proteins adsorbed on octyl-agarose showed the presence of only one band corresponding to a molecular weight of approximately 30 kDa (Figure 2). The bound lipase (lipase RT-1) was removed from the support by desorption with different concentrations of the detergent Triton X-100. The majority of lipase was obtained by desorption with 0.2% Triton X-100. 

Because a high detergent concentration is required for protein desorption from a hydrophobic support, we studied the effect of different detergents on lipase activity prior to purification from crude LG extracts.

The relative effects of detergents were assessed by comparing the enzymatic activity in the presence of detergent with the maximal enzymatic activity (Table 1). Tween 20 and Tween 80 belong to the same detergent series, and their effect on lipase activity was similar. The lipase showed maximal activity with Tween 20 and Tween 80 at concentrations ranging from 0.005% to 0.01%. Triton X-100 partially inhibited activity of the lipase at a concentration of about 0.01%. At concentrations of 0.25%, the lipase was markedly inhibited by all the detergents tested. The catalytic behavior of the enzymes depended strongly on the detergent concentration in the medium. For all detergents tested, low concentrations enhanced the activity of lipase from the LG of 2-day-old males toward *p*-NPL; at higher detergent concentrations, the activity declined and leveled off close to the value measured in the absence of detergent. For these reasons, we removed detergents from purified lipase samples by ultrafiltration prior to assessing enzymatic activity. 

**Table 1:** Effects of increasing concentrations of Tween 20, Tween 80, and Triton X-100 on the activity of lipase BT-1 isolated from the labial gland of 2-day-old *B. terrestris* males.

Detergent concentration [%]	Enzyme activity [%]*		
	Tween 20	Tween 80	Triton X-100
0.250	23.0	34.3	35.3
0.100	87.0	87.0	61.0
0.050	107.6	93.7	73.3
0.010	110.2	118.6	115.6
0.005	115.3	106.7	111.2
0.001	109.2	83.4	111.5

* The enzyme activity in the presence of detergents is expressed relative to the activity in detergent-free buffer (100%).

### Sequence analysis of lipase isolated from bumblebee labial glands

**Figure 3:**
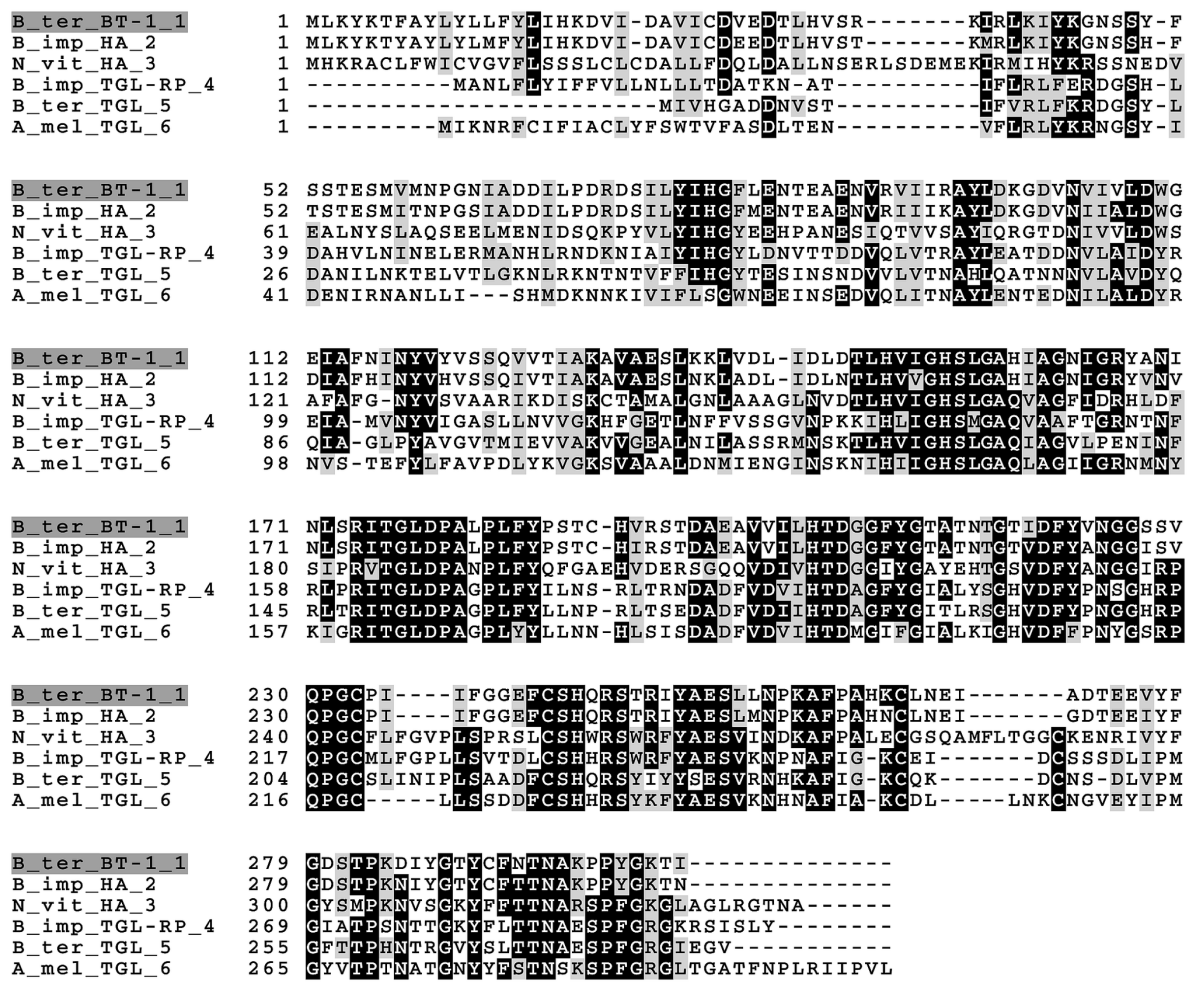
Alignment of lipase BT-1 from *B. tereestris* and lipases from other hymenopteran species. Protein sequence alignment was performed using the Multalin version 5.4.1 (Multiple sequence alignment with hierarchical clustering [http://multalin.toulouse.inra.fr/]. Identical sequence are marked by black background, similar sequence are on a grey background. The sequences of following lipases were compared: KF006994 (lipase member HA-like, *B. terrestris*), XP_003492427 (lipase HA-like, *B. impatiens*), XP_001599078 (lipase HA-like, *N. vitripennis*), XP_003487920 (pancreatic lipase-related protein 2-like, *B. impatiens*), XP_003398840 (pancreatic triacylglycerol lipase-like, *B. terrestris*), XP_001122903 (pancreatic triacylglycerol lipase-like, *A. mellifera*).

We used an MS-based proteomics approach using the NCBI-Bombus database to identify the sequence of the active protein isolated from bumblebee LGs. Eighteen peptides in the spectrum (Figure S1, Table S1) were homologous to the Genbank sequence for predicted *B. terrestris* lipase HA (*B. terrestris*, KF006994) and shared 88% similarity with the predicted lipase HA from *B. impatiens* (XP_003492427). It was also identical to the sequence constructed from the translated contigs obtained from RNA-seq data of the LG of *B. terrestris* [34]. We named this isolated lipase as BT-1 lipase. The sequence analysis indicated that lipase BT-1 is representative of neutral lipases [35] and contains also sequences typical for the pancreatic-like lipases. Because the lipase we isolated is the first example of neutral lipase from the cephalic part of the *B. terrestris* LG, its protein sequence was compared with those of known hymenopteran lipases: *B. terrestris*, *B. impatiens*, *Nasonia vitripennis* [36], and *Apis mellifera* [37] (Figure 3). Lipase BT-1 shares 36-100% identity with five active hymenopteran lipases (Table 2).

**Table 2:** Similarity in amino acid sequences of lipase BT-1 and its closest homologues.

Accession n.	Protein names (predicted)	Organism	I	S	E
KF006994	lipase member HA-like	*B. terrestris*	100	24	0.0
XP_003492427	lipase member HA-like	*B. impatiens*	88	574	0.0
XP_001599078	lipase member HA-like	*N. vitripennis*	41	215	4e-64
XP_003487920	pancreatic lipase-related protein 2-like	*B. impatiens*	36	194	2e-56
XP_003398840	pancreatic triacylglycerol lipase-like	*B. terrestris*	38	177	3e-50
XP_001122903	pancreatic triacylglycerol lipase-like	*A. mellifera*	36	177	9e-50

I [%] – identity, S – score, E – value

**Figure 4:**
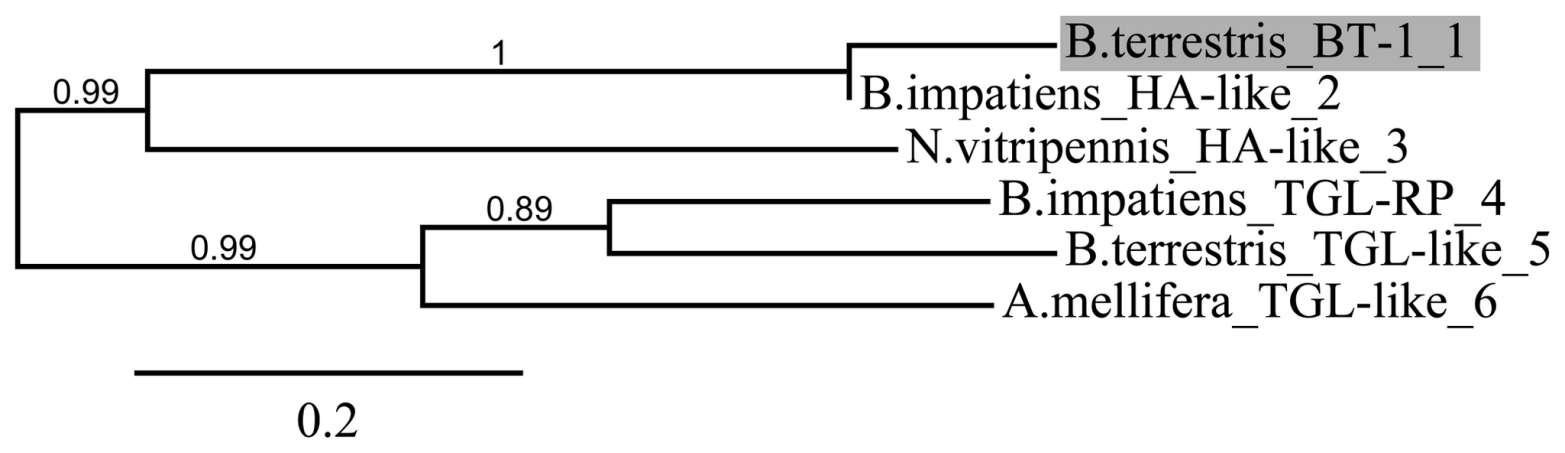
Phylogenetic analysis of lipase BT-1 from *B. terrestris* and its homologues. Phylogenetic analysis was performed with http://www.phylogeny.fr/version2_cgi/index.cgi. Accession numbers are as follows: 1 – KF006994 (lipase member HA-like, *B. terrestris*), 2 – XP_003492427 (lipase HA-like, *B. impatiens*), 3 – XP_001599078 (lipase HA-like, *N. vitripennis*), 4 – XP_003487920 (pancreatic lipase-related protein 2-like, *B. impatiens*), 5 – XP_003398840 (pancreatic triacylglycerol lipase-like, *B. terrestris*), 6 – XP_001122903 (pancreatic triacylglycerol lipase-like, *A. mellifera*).

A phylogenetic tree shows the potential evolutionary relationship of lipase BT-1 with other members of the triacylglycerol lipase family (Figure 4). Lipase BT-1 is most similar to lipase from *B. impatiens* and more distantly related to the triacylglycerol lipases found on neighboring branches of the tree.

### Lipase BT-1 prefers medium-length fatty acid ester substrates

To determine the substrate specificity of lipase BT-1, we tested its ability to hydrolyze *p*-nitrophenyl esters with different chain lengths [*p*-NPC (C8), *p*-NPD (C10), *p*-NPL (C12), *p*-NPP (C16), *p*-NPS (C18)].

**Figure 5:**
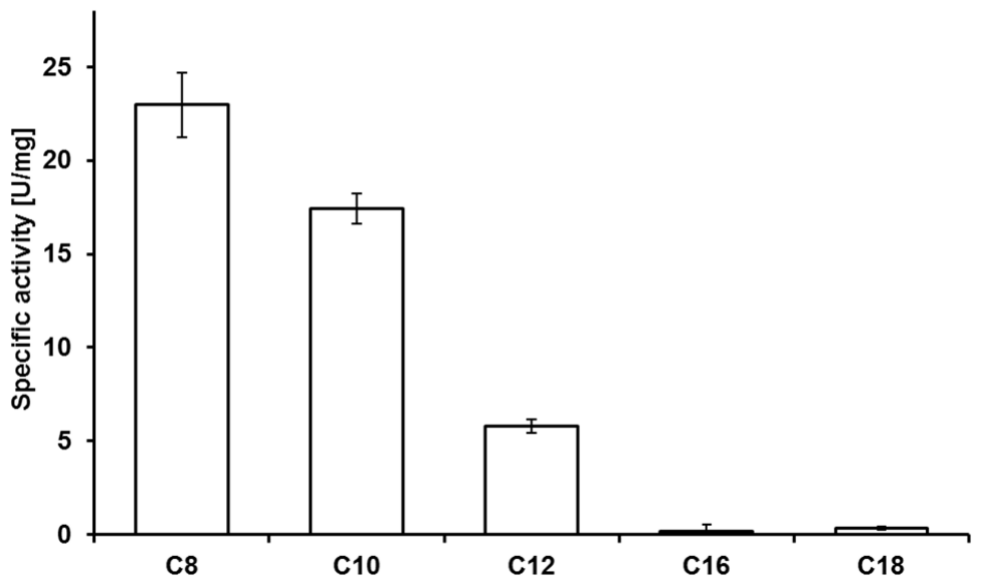
Substrate specificity of lipase from 2-day-old *B. terrestris* towards various *p*-nitrophenyl esters. The following substrates were used at a concentration of 0.3 mM: *p*-NPC (C8), *p*-NPD (C10), *p*-NPL (C12), *p*-NPP (C16), *p*-NPS (C18).

The enzyme displayed activity towards a broad range of acyl chain lengths, but maximal activity was observed with medium-length fatty acid esters. The enzyme hydrolyzes *p*-NPD and *p*-NPL substrates (C10 and C12, Figure 5), although not as well as the *p*-NPC substrate (C8). The enzyme hydrolyses *p*-NPS and *p*-NPP as well but with low chemical yield. This suggests that the enzyme we isolated is indeed a lipase than rather an esterase because esterases prefer short-chain acyl esters (less than 10 carbon atoms). The classification of the enzyme as a lipase is further supported by its lack of activity against emulsions of slightly water-soluble short-chain triacylglycerols such as tripropionin and tributyrin [38]. Lipase substrates are typically long-chain (C10 and longer) fatty acid esters available in micellar form [39]. We chose *p*-NPL (C12) as lipase BT-1 substrate for further studies.

### Lipase BT-1 follows Michaelis-Menten kinetics

To study the enzyme-substrate affinity, we measured the kinetic parameters of BT-1 lipase with concentrations of *p*-NPL ranging from 0.05 mM to 0.4 mM. The resulting Lineweaver-Burk plots were linear, indicating that the hydrolysis of *p-*NPL by BT-1 lipase follows Michaelis-Menten kinetics. K_m_ and V_max_ values were calculated from the Lineweaver-Burk plot (Table 3).

**Table 3:** Specific activity, K_m_, and V_max_ values for hydrolysis of *p-*NPL substrate by lipase BT-1.

Specific activity [U/mg]	0.0058 ± 0.0002
K_m_ [mM]	0.0011 ± 0.0003
V_max_ [U/mg]	0.15 ± 0.01
V_max_/K_M_	136

Activity values are the mean of three independent assays. K_m_ and V_max_ values were calculated from the Lineweaver-Burk plot

### Effects of temperature and pH on lipase activity

We found that the optimal reaction temperature for the activity of purified lipase rom bumblebee LGs towards *p*-NPL is 50°C (Figure 6A). At this temperature, the lipase showed a 2-fold increase in activity relative to its behavior at 25°C. A drop in activity was observed at temperatures higher than 55°C. 

**Figure:**
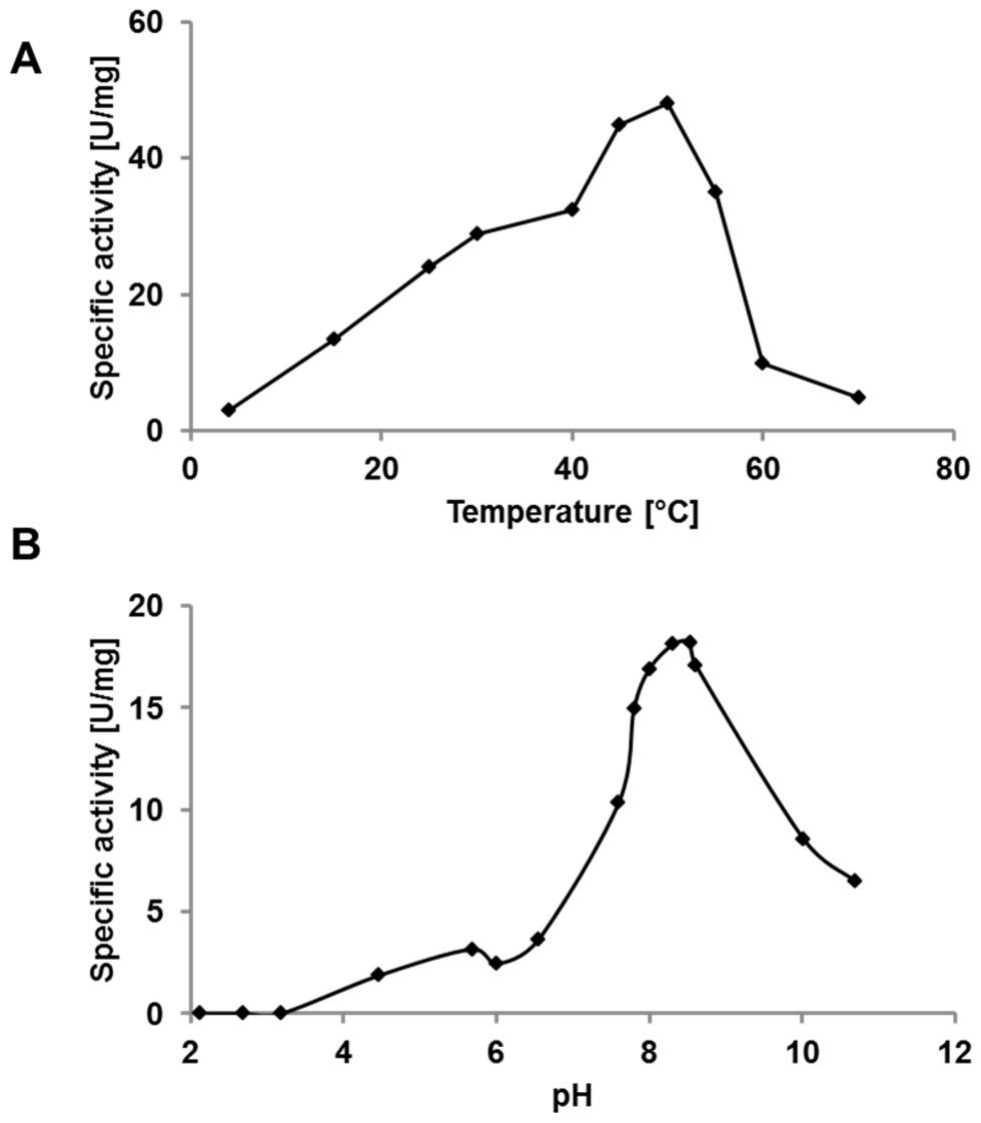
Figure 6. A. Effect of temperature on the activity of lipase BT-1. The lipase BT-1 incubated in in 50 mM Tris-HCl buffer (pH 8) at various temperatures (4-70°C) and assayed for lipase activity. **B. Effect of pH on the activity of lipase BT-1.** The lipase BT-1 incubated in various buffer (pH 2.2-11.0) at 25°C and assayed for lipase activity.

We also tested the lipase activity at various pH values at 25°C with *p*-NPL as substrate. We determined that the enzyme has a pH optimum of approximately 8.3 (Figure 6B). 

### Metallic cations and other enzyme inhibitors affect the activity of the purified lipase

We found that 1.0 mM Cu^2+^, Hg^2+^, Ag^2+^, and Fe^3+^ inhibited the purified lipase, suggesting that they can alter the enzyme conformation [40]. Of these cations, Fe^3+^ displayed the strongest inhibitory effect, showing potent inhibition at 0.1 mM concentration (Table 4). Cation concentrations higher than 10 mM significantly inhibited lipase activity. Inhibition by Hg^2+^ may indicate the importance of thiol-containing amino acid residues in the enzyme function [41]. 

**Table 4:** Effect of different cations and enzyme inhibitors on the activity of purified lipase BT-1.

Inhibitor	Relative activity of lipase [%]		
	0.1 mM	1 mM	10 mM
Na^+^	145.0 ± 1.0	130.7 ± 0.9	123.2 ± 0.4
Ca^2+^	176.2 ± 1.2	135.3 ± 0.7	85.1 ± 0.3
Mg^2+^	135.8 ± 0.4	179.1 ± 0.6	150.3 ± 0.7
K^+^	166.8 ± 1.7	127.2 ± 0.4	78.7 ± 0.6
Zn^2+^	117.7± 0.2	68.6 ± 0.1	66.8 ± 0.4
Mn^2+^	131.1 ± 0.6	151.4 ± 0.5	81.5 ± 0.3
Cu^2+^	76.3 ± 0.4	51.9 ± 0.4	22.8 ± 0.5
Co^2+^	147.0 ± 1.2	164.8 ± 1.8	107.2 ± 0.9
Ni^2+^	170.1 ± 1.1	192.2 ± 1.5	143.5 ± 0.9
Ag^+^	80.1 ± 0.8	0.0	0.0
Ba^2+^	104.6 ± 1.1	108.2 ± 1.3	98.8 ± 0.9
Hg^2+^	54.5 ± 0.6	41.3 ± 0.4	22.3 ± 0.6
NH_4_^+^	115.6 ± 1.9	114.9 ± 0.6	112.5 ± 0.3
Fe^3+^	0.0	0.0	0.0
NaEDTA	174.6 ± 1.5	91.8 ± 0.6	49.2 ± 0.3
urea	143.1 ± 0.6	120.1 ± 0.6	92.2 ± 0.7
SDS	56.9 ± 0.3	55.6 ± 1.0	44.8 ± 1.1
buffer	100.0 ± 0.1	100.0 ± 0.2	100.0 ± 0.6

The remaining activity [%] is expressed relative to the control (lipase activity in buffer alone). Data are given as means ± S.D., *n* = 3.

We tested the effects of additional potential inhibitors on the activity of the purified lipase (Table 4). The presence of the chelating agent NaEDTA did not affect the enzyme activity at concentrations ranging from 0.1 to 1 mM, suggesting that the purified enzyme is not a metalloenzyme [42]. On the other hand, 10 mM NaEDTA reduced the activity by 50%. Urea had a positive effect on the lipase activity at all concentrations tested. The addition of SDS had an inhibitory effect on the lipase, indicating that the protein conformation may be affected by the presence of SDS.

### Substrate competition reactions

Chain-length selectivity profiles of lipase BT-1 were determined by multiple substrate competition for hydrolysis of triacylglycerols and diacylglycerols. All triacylglycerol acyl chains were saturated and had an even-number carbon chain. We inferred the enzyme’s substrate specificity from the effects of addition of non-radiolabeled competing substrates on the rate of hydrolysis of radiolabeled trioleate. In this experimental set-up, substrates more effective than trioleate should reduce the amount of radioactivity recovered in the assay. In competition reactions between triacylglycerols (trimyristate, tripalmitate, tristearate, and radiolabeled trioleate), hydrolysis preferentially occurred in the order tristearate > tripalmitate > trimyristate (Figure 7A). Lipase preferentially hydrolyzed trioleate in the presence of tristearate, while trimyristate was a less effective substrate than trioleate. 

**Figure:**
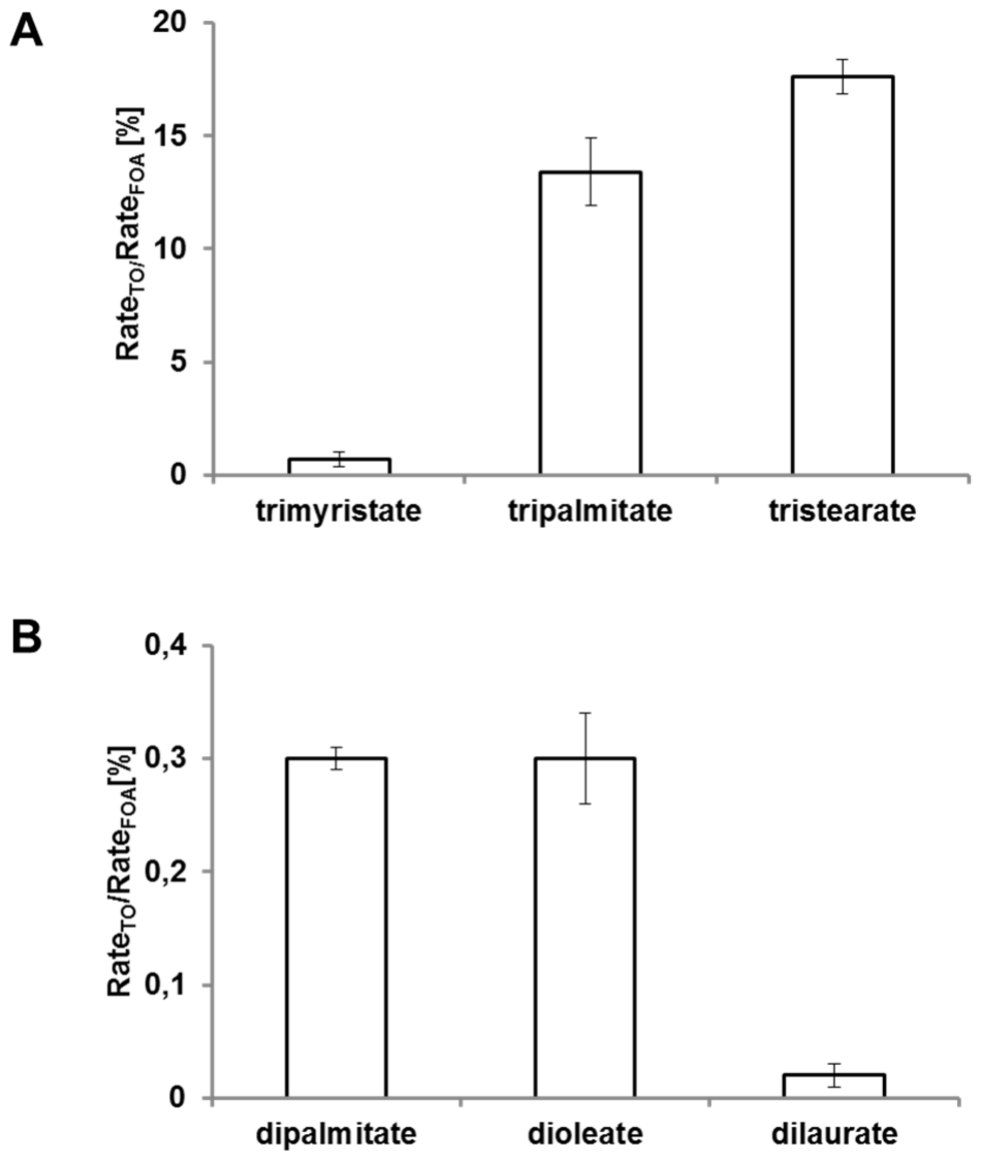
Figure 7. A. Effect of triacylglycerols on rates of oleic acid released from radiolabeled triolein by lipase BT-1. This assay employed ^14^C-labeled triolein as a substrate, and activity was measured as radioactivity recovered in free fatty acid after exposure to lipase. **B. Effect of diacylglycerols on rates of oleic acid released from radiolabeled triolein by lipase BT-1.** This assay employed ^14^C-labeled triolein as a substrate, and activity was measured as radioactivity recovered in free fatty acid after exposure to lipase.

The rate of the enzymatic reaction with diacylglycerols used as competitive substrates (dipalmitate, dioleate, dilaurate, and radiolabeled dioleate) was 10-fold lower than that of the reaction with triacylglycerols. In the presence of dilaurate and dipalmitate, the hydrolytic rate was higher than that of the reaction with dioleate (Figure 7B). Dioleate had a negative effect on hydrolysis of radiolabeled trioleate. 

### Age-dependent analysis of gene expression using qPCR

**Figure 8:**
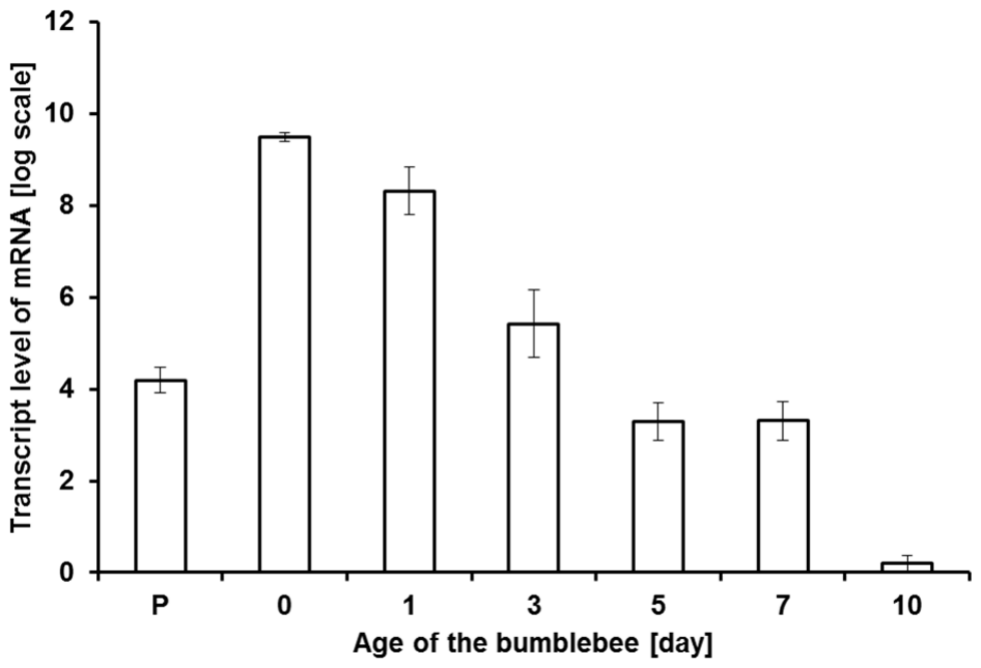
Relative gene expression levels of lipase gene in labial glands from *B. terrestris* males of different ages quantified by qPCR. (P: pharate). Data are shown as mean ± SD of three biological replicates.

We studied the relative expression levels of the lipase RT-1 gene in LGs of *B. terrestris* males of different ages. We detected an approximately 40-fold increase in the level of lipase mRNA in the LGs from pharate males compared to newborn males (Figure 8). The mRNA transcript level in 1-day-old bumblebees was modestly lower than that found in newborn males. We observed a significant decrease in the LG lipase transcript level between 1-day-old and 5-day-old males. We detected relatively constant lipase mRNA levels in LG from older *B. terrestris* males, except in the case of 10-day-old males, where we found a reduced transcript level compared to 7- and 12-day-old bumblebees. Analysis of RNA-seq data of the LG of *B. terrestris* [34] indicated that the lipase transcript level is 852 times higher in the labial gland than in the fat body. 

## Discussion

Very little is known about insect lipases, although their presence and substrate specificity in different insect tissues including the midgut, fat body and Hymenopteran venon have been investigated [5,10,11,43]. To develop a thorough understanding of the metabolism and transport of lipids in insects, the enzymes involved must be characterized. 

In this work, we found that lipase isolated from the cephalic part of labial pheromone glands of *B. terrestris* males possesses high hydrolytic activity against *p*-nitrophenyl esters with different acyl chain lengths and a low but significant lipolytic activity against trioleate. To confirm that all the activities observed were produced by the same enzyme, we purified the lipase to homogeneity. 

In the present study the expression of the lipase RT-1 gene obtained by qPCR correlated well with the analytical measurements of age-dependent dynamic changes in lipase activity. The level of lipase mRNA in the LGs from newborn males was the highest and maximal lipase activity was observed in LGs from 3-day-old individuals, which corresponds with the timing of pheromone formation in the LG [43].

Enzyme function is efficient only within limited temperature and pH ranges. Most insects have enzymes with a pH optimum of 6–7, but in caterpillars, optimal activity occurs at about pH 10 [44]. The lipase BT-1 showed strong activity at pH 8, but activity decreased rapidly at pH values exceeding 10. These data are in agreement with the characteristics of lipases from *Manduca sexta* and *Lymantria dispar* [5,45]. The activity of crude lipase increases with temperature, but at higher temperatures, the enzymes become denatured. Consequently, activity rises to a maximum and then declines as the rate of denaturation increases.

Insect lipases vary widely in molecular mass. For example, the major triglyceride lipase from the fat body of *M. sexta* has a molecular weight of 76 kDa [5], a novel lipase from *Cephaloleia presignis* (Coleoptera: Chrysomelidae) is approximately 30 kDa [8], a lipase with antiviral activity from isolated from the digestive juice of *B. mori* is 28 kDa (according to MALDI) [45], and the newly identified BT-1 lipase from the *B. terrestis* LG is approximately 30 kDa.

Mrdacovic et al. [46] found that Ca^2+^ non-significantly decreased the enzyme activity of gypsy moth midgut lipase, suggesting that the activity is Ca-independent. Phospholipases from *Nicrophorus marginatus* (Coleoptera) [47] and *Cochliomyia hominivorax* (Diptera) [48] are Ca-dependent, while the phospholipase of *Aedes aegypti* (Diptera) is Ca-independent [49]. Our results indicate that lipase BT-1 from *B. terrestris* is Ca-dependent.

To our knowledge, this is the first report on neutral lipase from the cephalic part of *B. terrestris* labial glands. Purified lipase BT-1 displays broad specificity but preferentially hydrolyzes shorter fatty acid chains (C8-C12) from diacylglycerols and triacylglycerols. The lipase is most active at neutral pH, with a pH optimum of 8.3. The highest level of lipase BT-1 gene and protein expression is in young individuals. In bumblebees older than 3 days, the lipase gene expression level and enzymatic activity are decreased. Interestingly, according to transcript-level RNA seq analysis, the expression of lipase BT-1 is significantly higher in the bumblebee LG than in the fat body. The lipase BT-1 gene is the second most abundantly expressed gene in the labial gland of *B. terrestris*, which suggests important role of this enzyme in this tissue where pheromones are accumulated. 

Taken together, our results suggest that lipase BT-1 might participate in the release of shorter fatty acids from diacylglycerols transported by hemolymph to the LG from storage triacylglycerols. These fatty acids might be further modified by specific enzymes (desaturases, reductases, etc.) into pheromone components. 

## Supporting Information

Figure S1 **Predicted amino acid sequence of lipase BT-1 from the labial gland of *B. terrestris*.** Matching peptides found by MS are shown in grey. According to http://prosite.expasy.org/scanprosite/ , the residues 149-158 (LHVIGHSLGA) comprise the active site. Ser 155, Asp 179, and His 244 form a catalytic triad (http://www.ncbi.nlm.nih.gov/Structure/cdd/cdd.shtml).
(TIF)

Table S1 **Parameters of matched peptides obtained by mass spectrometric analysis (lipase BT-1 from *B. terrestris*).**
(DOC)
