# Supplementary figures and images for: Characterization of Neutral Lipase BT-1 Isolated from the Labial Gland of Bombus terrestris Males
Source: PLoS One. 2013 Nov 8;8(11):e80066. doi: 10.1371/journal.pone.0080066 (PMC3832651; doi:10.1371/journal.pone.0080066)

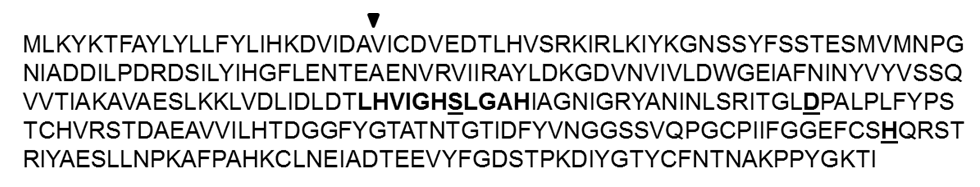

Supplement: Figure S1 — Predicted amino acid sequence of lipase BT-1 from the labial gland of B. terrestris. Matching peptides found by MS are shown in grey. According to http://prosite.expasy.org/scanprosite/ , the residues 149-158 (LHVIGHSLGA) comprise the active site. Ser 155, Asp 179, and His 244 form a catalytic triad (http://www.ncbi.nlm.nih.gov/Structure/cdd/cdd.shtml). (TIF) [file pone.0080066.s001.tif]
