# Supplementary material for: Characterization of Neutral Lipase BT-1 Isolated from the Labial Gland of Bombus terrestris Males
Source: PLoS One. 2013 Nov 8;8(11):e80066. doi: 10.1371/journal.pone.0080066 (PMC3832651; doi:10.1371/journal.pone.0080066)
Supplement: Table S1 — Parameters of matched peptides obtained by mass spectrometric analysis (lipase BT-1 from B. terrestris). (DOC) [file pone.0080066.s002.doc]

**Table S1.** Parameters of matched peptides obtained by mass spectrometric analysis (lipase BT-1 from *B. terrestris*)

| Peptides | DeltaM (ppm) | Score XC |
| --- | --- | --- |
| DIYGTYCFNTNAKPPYGK | 0.0015 | 18 |
| DSILYIHGFLENTEAENVR | 0.0008 | 24 |
| ITGLDPALPLFYPSTCHVR | 0.0016 | 24 |
| IYAESLLNPK | -0.0007 | 16 |
| LVDLIDLDTLHVIGHSLGAHIAGNIGR | 0.0131 | 13 |
| VICDVEDTLHVSR | 0.0027 | 18 |
| DIYGTYCFNTN | -0.0060 | 16 |
| IADDILPDR | -0.0013 | 12 |
| CLNEIADTEEVYFGDSTPK | 0.0002 | 27 |
| DSILYIH | -0.0015 | 6 |
| SLGAHIAGNIGR | 0.0007 | 10 |
| CLNEIADTEEVYFGDSTPK | -0.0027 | 30 |
| DIYGTYCFNTN | -0.0060 | 11 |
| DSILYIHGFLENTEAENVR | 0.0008 | 26 |
| ITGLDPALPLFYPSTCHVR | 0.0016 | 22 |
| IYAESLLNPK | -0.0007 | 16 |
| LVDLIDLDTLHVIGHSLGAHIAGNIGR | 0.0191 | 15 |
| VICDVEDTLHVSR | 0.0027 | 14 |
